# Interspecies Interactions Between Streptococcus Mutans and Streptococcus Agalactiae *in vitro*

**DOI:** 10.3389/fcimb.2020.00344

**Published:** 2020-07-07

**Authors:** Tingjun Liu, Jia Liu, Jianwei Liu, Ruiqi Yang, Xianjun Lu, Xuesong He, Wenyuan Shi, Lihong Guo

**Affiliations:** ^1^Hospital of Stomatology, Guanghua School of Stomatology, Sun Yat-sen University, Guangzhou, China; ^2^Guangdong Provincial Key Laboratory of Stomatology, Guangzhou, China; ^3^The Forsyth Institute, Cambridge, MA, United States; ^4^Department of Oral Medicine, Infection and Immunity, Harvard School of Dental Medicine, Boston, MA, United States

**Keywords:** group B *Streptococcus*, coaggregation, *Streptococcus mutans*, interspecies interactions, *Streptococcus agalactiae*

## Abstract

*Streptococcus mutans* is an oral species closely associated with dental caries. As an early oral colonizer, *S. mutans* utilizes interspecies coaggregation to promote the colonization of subsequent species and affect polymicrobial pathogenesis. Previous studies have confirmed several adhering partner species of *S. mutans*, including *Candida albicans* and *Fusobacterium nucleatum*. In this study, we discovered new intergeneric co-adherence between *S. mutans* and the saliva isolate *Streptococcus agalactiae* (GBS-SI101). Research shows that GBS typically colonizes the human gastrointestinal and vaginal tracts. It is responsible for adverse pregnancy outcomes and life-threatening infections in neonates and immunocompromised people. Our results revealed that GtfB and GtfC of *S. mutans*, which contributed to extracellular polysaccharide synthesis, promoted coaggregation of *S. mutans* with GBS-SI101. In addition, oral streptococci, including *Streptococcus sanguinis, Streptococcus gordonii* and *S. mutans*, barely inhibited the growth of GBS-SI101. This study indicated that *S. mutans* could help GBS integrate into the *Streptococcus-*associated oral polymicrobial community and become a resident species in the oral cavity, increasing the risk of oral infections.

## Introduction

The oral microbiome consists of ~700 species of bacteria (Paster et al., 2010). The disparity of oral bacteria among different individuals is relatively low (The Human Microbiome Project Consortium, 2012). In contrast, the complexity of the oral microbiota in a single individual is the highest compared to that of other body sites (Aas et al., 2005). Oral microbes engage in extensive cell-cell interactions. Early colonizers adhere to accessible host surfaces and facilitate the colonization of subsequent species via intergeneric coaggregation, forming highly structured polymicrobial oral biofilms (Kolenbrander et al., 2010). In addition, intergeneric cell-cell communication can trigger signaling cascades and induce changes in the gene expression of the attached species, influencing the expression of virulence factors of microorganisms in the oral cavity (Krzysciak et al., 2014).

Oral streptococci, including *Streptococcus mutans* (*S. mutans*), are the earliest oral colonizers, which can be acquired immediately after birth (Abranches et al., 2018). *S. mutans* is a prevalent etiological bacterial species associated with early childhood caries (ECC) (Van Houte et al., 1982; Tanzer et al., 2001; Agnello et al., 2017). Accumulative reports show that *S. mutans* has been detected in predentate infants, and the colonization rate of *S. mutans* increases rapidly as primary teeth erupt (Wan et al., 2001, 2003; Plonka et al., 2012). Infants usually acquire *S. mutans* from their mothers, and maternal salivary bacterial abundance is closely associated with early oral infections among children (Chaffee et al., 2014). *S. mutans* possesses multiple virulence factors that contribute to tooth decay. Potent in synthesizing extracellular polysaccharides (EPS), *S. mutans* adheres to enamel surfaces and forms intercellular clustering within dental plaques. *S. mutans* can metabolize digestible carbohydrates to create an acidic microenvironment (acidogenicity), which leads to enamel demineralization, meanwhile, *S. mutans* can thrive under low pH conditions (aciduricity) (Lemos and Burne, 2008; Forssten et al., 2010).

The ability of primary oral inhabitants to bind to various subsequent species has been well established (Hojo et al., 2009), but there are limited reports on the interspecies co-adherence between *S. mutans* and other species. Previous studies show that *S. mutans* adheres to *Candida albicans* (*C. albicans*), and the presence of *C. albicans* enhances the production of the EPS-rich matrix in dual-species biofilms (Barbieri et al., 2007; Metwalli et al., 2013; Falsetta et al., 2014). Guo et al. (2017) discovered that *S. mutans*, utilizing its adhesin SpaP, specifically binds to RadD of *Fusobacterium nucleatum ssp. polymorphum (Fnp)*. The interplay between *S. mutans* and *Fnp* improves both species' abilities to effectively colonize the oral cavity (Guo et al., 2017). In this study, using a pull-down assay, we identified a *Streptococcus agalactiae* (*S. agalactiae*, also known as Group B *Streptococcus*, GBS) strain from human saliva samples, which displayed direct physical interactions with *S. mutans*.

GBS is a facultative anaerobe and a leading cause of infections in pregnant women and neonates (Gibbs et al., 2004; Kim, 2010). Half of GBS infections in pregnant women affect the upper genital tract, placenta, or amniotic sac, increasing the risk of fetal death (Phares et al., 2008). A mother who carries GBS can transmit it to her infant during delivery. GBS infections in neonates often cause meningitis and can lead to long-term neurodevelopmental impairment after recovery (Phares et al., 2008; Kohli-Lynch et al., 2017). GBS infections are also common among immunocompromised individuals and the elderly, causing bacteremia, soft tissue infections, or pneumonia (Farley and Strasbaugh, 2001; High et al., 2005). The gastrointestinal and vaginal tracts are the main reservoirs for GBS (Meyn et al., 2009). Some studies have reported the presence of GBS in the oropharynx (Hickman et al., 1999; Van der Mee-Marquet et al., 2008).

While adherence via pili to salivary pellicles could promote GBS colonization within the oral cavity (Brittan and Nobbs, 2015), an alternative mechanism allowing GBS to persist within oral cavity could be due to its ability to integrate into existing biofilms through intergeneric coaggregation, as has been well documented in many other bacteria (Jakubovics et al., 2014). However, there is scarce evidence to demonstrate the direct physical interactions between GBS and oral bacteria. Thus, little is known regarding the role of oral species in GBS's survival and persistence within the oral microbial community.

This study aimed to investigate the interactions between *S. mutans* and GBS and evaluate the potential for *S. mutans* to facilitate GBS integration into the *Streptococcus*-associated oral multispecies community.

## Materials and Methods

### Bacterial Strains and Growth Conditions

*S. mutans* UA140, *Streptococcus sanguinis* (*S. sanguinis*) ATCC 10,556 and *Streptococcus gordonii* (*S. gordonii*) DL1 were grown in Todd-Hewitt broth (THB) (BD Difco, Detroit, MI, USA) under anaerobic conditions (10% H_2_, 10% CO_2_, 80% N_2_) at 37°C. Professor Wenyuan Shi's laboratory has preserved *gtfB*-, *gtfC*-, *gtfD*-, *gtfBC*-, and *gtfBCD*-deficient strains of *S. mutans* (kindly provided by H. Kuramitsu, University at Buffalo, State University of New York, NY, USA).

SHI medium, which can support the growth of a highly diverse oral microbial community *in vitro* (Tian et al., 2010), was adopted to cultivate the saliva microbiota. SHI medium has the following composition (Tian et al., 2010): proteose peptone (Difco) 10 g/L; trypticase peptone (Difco) 5.0 g/L; yeast extract (Difco) 5.0 g/L; KCl 2.5 g/L; sucrose 5 g/L; haemin 5 mg/L; VitK 1 mg/L; urea 0.06 g/L, arginine 0.174 g/L; mucin (type III, porcine, gastric, Sigma Chemical Co., St. Louis, Mo) 2.5 g/L; sheep blood (Colorado serum company) 5% and N-acetylmuramic acid (NAM) 10 mg/L.

### Saliva Collection

Saliva samples were collected from 10 healthy participants aged 25~40. None of the participants was being treated for any systemic disease or dental disease or taking any medication. Participants were asked to refrain from any food or drink 2 h before saliva donation. At 10 a.m., participants were asked to spit directly into the saliva collection tubes, and 5 ml of saliva was collected from each participant.

### *S. mutans* Biofilm Formation

One milliliter of saliva from each participant was pooled together and centrifuged at 14,000 × g for 10 min at 4°C to remove saliva microorganisms. The supernatant, referred to as pooled saliva proteins, was collected to coat sterile 6-well flat-bottomed polystyrene microtiter plates (Corning, New York, NY). The 6-well culture plates were dried and sterilized under UV light for 1 h before bacterial inoculation. Overnight culture (OD_600_~ 0.7) of *S. mutans* was diluted 1:100 into THB containing 0.5% (w/v) sucrose, with a final concentration of approximately 2 × 10^5^ cells/ml. A total of 400 μl of this suspension was inoculated into each well and incubated overnight under anaerobic conditions for biofilm formation. The wells were washed three times with phosphate-buffered saline (PBS) to remove planktonic and loosely bound *S. mutans* cells.

### Cultivating Human Saliva-Derived Microbiota (S-Mix)

Pooled saliva was centrifuged at 2,600 × g for 10 min at 20°C to spin down large debris and eukaryotic cells. The supernatant was inoculated into 5 ml of SHI medium and incubated anaerobically overnight to obtain saliva-derived microbiota (S-mix). S-mix was used in the following experiments in this study.

### Pull-Down Assay

To identify microbial species from saliva samples that could directly adhere to *S. mutans*, we performed a pull-down assay. The S-mix was resuspended in sterile coaggregation buffer (CAB) (OD_600_~1). CAB consists of 150 mM NaCl, 1 mM Tris, 0.1 mM CaCl_2_, and 0.1 mM MgCl_2_ (Cisar et al., 1979). A total of 400 μl of S-mix was overlaid onto the 6-well culture plate with pre-existing *S. mutans* biofilms and incubated under anaerobic conditions for 1, 2, and 3 h, respectively. The wells were then rinsed three times with PBS to remove cells that failed to adhere to *S. mutans* biofilms. Biofilm *S. mutans* cells with the remaining binding species (co-adhering mixtures) were carefully scraped off. The co-adhering mixtures, obtained after each incubation time, were divided into two portions. One portion was immediately subjected to extraction of total bacterial genomic DNA. The other portion was regrown in fresh SHI medium anaerobically overnight, followed by DNA extraction. Four hundred microliters of sterile CAB were added to 6-well culture plates with pre-coated pooled saliva proteins or pre-existing *S. mutans* biofilms to serve as control.

### PCR-DGGE Analysis

The co-adhering mixtures were added directly into a 0.5-mL screw cap microtube containing lysis buffer (MasterPure^TM^ DNA Purification Kit, Epicenter) and 0.1-mm silica beads. The samples were treated with bead beating for 30 s three times at 1-min intervals at 4°C. After centrifugation of the samples at 13,000 × *g* for 5 min, the supernatant was transferred to a fresh tube and incubated with Proteinase K for 1 h at 56°C. The total genomic DNA of samples from each group was isolated using the MasterPure^TM^ DNA Purification Kit according to the manufacturer's instructions. DNA quality and quantity were determined using a Spectronic Genesys UV spectrophotometer at 260 nm and 280 nm (Spectronic Instrument, Inc. Rochester, New York, USA). Amplification of bacterial 16S rRNA genes by PCR was carried out as described previously (Li et al., 2005; He et al., 2011). Bac1 (5′- CGCCCGCCGCGCCCCGCGCCCGTCCCGCCGCCCCCGCCCGACTACGTGCCAGCA GCC-3) and Bac2 (5′-GGACTACCAGGGTATCTAATCC-3′) (Sheffield et al., 1989) were used as the universal primer set to amplify an approximately 300-bp internal fragment of the 16S rRNA gene. Each 50 μl PCR contained 100 ng of genomic DNA, 200 μm of each dNTP, 40 pmol of each primer, 4.0 mM MgCl_2_, 5 μl 10× PCR buffer, and 2.5 U Taq DNA polymerase (Invitrogen). Cycling conditions were set as follows: 94°C/3 min, 30 cycles of 94°C/1 min, 56°C/1 min and 72°C/1 min, and 72°C/5 min (final extension period).

Polyacrylamide gels (8%) with a denaturing gradient between 40 and 70% (urea/formamide) were prepared as previously described (Tian et al., 2010). Each well was loaded with 300 ng of the PCR product. The gels were submerged in 1 × TAE (Tris-acetate-EDTA) buffer, and PCR products were separated by electrophoresis for 16 h at 60°C using a fixed voltage of 60 V (Bio-Rad DCode System, Bio-Rad, Hercules, CA, USA). After electrophoresis, the gels were rinsed and stained for 15 min in 1 × TAE buffer containing ethidium bromide (0.5 μg/ml), followed by destaining in 1 × TAE buffer for 10 min. DGGE profile images were recorded using the Molecular Imager Gel Documentation system (Bio-Rad, Hercules, CA, USA). Diversity Database Software (Bio-Rad, Hercules, CA, USA) was used to assess the intensity of bands of interest.

### Identification of Bacterial Species From DGGE Gel

The DNA bands, which only existed in co-adhering mixtures or S-mix but not in the control, were excised from the DGGE gels, transferred to 1.5 ml microfuge tubes containing 20 μl of sterile H_2_O, and kept overnight at 4°C to allow DNA recovery.

Procedures for species identification were performed as described previously with modifications (He et al., 2011). DNA samples were reamplified with Bac1 and Bac2, purified using the QIAquick PCR purification kit (Qiagen), and sequenced. The obtained sequences were matched to nucleotide BLAST searches against the NCBI (http://blast.ncbi.nlm.nih.gov/) and Human Oral Microbiome Database (http://www.homd.org/index.php). The saliva isolate *S. agalactiae*, designated GBS-SI101, was identified by sequencing.

### Isolation and Identification of Bacterial Species Pulled Down by Biofilm *S. mutans* Cells

A 100-μl aliquot of co-adhering mixtures was taken, subjected to serial dilution, and seeded onto SHI agar plates supplemented with sheep blood, which helped detect β-hemolytic strains. The plates were incubated for 48 h under anaerobic conditions. Based on the sequencing result from DGGE gels, colonies that were β-hemolytic and grayish-white on a sheep blood-containing plate (Cools and Melin, 2017) were picked and grown in fresh SHI medium under anaerobic conditions until turbid. The bacterial genomic DNA was prepared using the MasterPure^TM^ DNA Purification Kit (Epicenter).

For species identification, the universal bacterial 16S rDNA primer pair 27F (5′- AGAGTTTGATCCTGGCTCAG-3′) and 1492R (5′-GGTTACCTTGTTACGACTT-3′) (Martinlaurent et al., 2001) was used to generate an approximately 1,500-bp amplicon. Procedures described previously were used to proceed with PCRs and sequence purified PCR products (He et al., 2011). Sequences with over 98% identity to those deposited in the databases were considered to be positive for taxa identification.

### Establishment of Dual-Species Biofilms

Overnight culture (OD_600_~ 0.7) of *S. mutans* was diluted 1:200 into THB containing 0.5% (w/v) sucrose. Four hundred microliters of cell suspension were inoculated onto 6-well culture plates with pre-coated pooled saliva proteins, and the plates were incubated anaerobically overnight to form *S. mutans* biofilms. Overnight culture (OD_600_~ 0.8) of GBS-SI101 was diluted 1:100 into THB, and 400 μl of GBS-SI101 was overlaid onto 6-well culture plates with pre-existing *S. mutans* biofilms or pre-coated pooled saliva proteins, followed by 16 h incubation under anaerobic conditions.

### Confocal Laser Scanning Microscopy (CLSM) and Image Analysis

Overnight dual-species biofilms were rinsed three times with PBS to remove the unattached bacteria. The average cell sizes for *S. mutans* bacterium and GBS bacterium are 0.5–0.75 μm and 0.6–1.0 μm, respectively (Nagao, 2015; Zhou and Li, 2015). For visualization, biofilms were stained with 1.67 μM SYTO 9 green-fluorescent nucleic acid stain (Life Technologies) according to the manufacturer's protocol. *S. mutans* was further labeled with a specific anti-*S. mutans* monoclonal antibody SWLA1–IgG2a, as described previously (Fang et al., 2005). The far-red dye Alexa Fluor 633-conjugated goat anti-mouse IgG (Sigma, St. Louis, MO) was used as a secondary antibody.

The biofilms were monitored through a 40× oil-immersion lens with a PASCAL LSM5 CLSM (Zeiss, Germany). Image stacks of five randomly chosen spots were collected for each experimental sample, and representative images are shown in the results section. The excitation/emission maxima for SYTO 9 staining were set at 488 nm/500 nm. A 560 nm and 650 nm long-pass emission filter was utilized to reveal Alexa Fluor 633-labeled cells within the biofilms. CLSM images were analyzed using the computer program COMSTAT.

### Fluorescence-Based Coaggregation Assay

To achieve a visual and sensitive readout, a fluorescence-based coaggregation assay was used with minor modifications (Wu et al., 2015). GBS-SI101 cells were grown in THB anaerobically, collected from exponential-phase culture, washed, and adjusted to an OD_600_ of 10 in CAB. Two hundred microliters of cell suspension were anaerobically stained with 1 μl of 10 μM SYTO 9 (Life Technologies) for 30 min at room temperature. Cells were washed 10 times with PBS and resuspended in CAB to a final OD_600_ of 1. In parallel, *gtfB*-, *gtfC*-, *gtfD*-, *gtfBC*-, and *gtfBCD*-deficient strains of *S. mutans* and the parent strain were collected from exponential-phase culture, washed, and resuspended in CAB to a final OD_600_ of 1.

For the coaggregation assay, *S. mutans* and GBS-SI101 were mixed with equal volumes of 250 μl in reaction tubes, vigorously vortexed for 30 s, and allowed to stand at room temperature for 10 min under anaerobic conditions. The coaggregation pellet was collected at 100 g for 1 min, and the supernatant with unbound bacterial cells was discarded carefully using a micropipette (Research Plus, Eppendorf, Germany). The coaggregation pellet was added to 5 ml of PBS, followed by 5 min of vortexing. Next, 250 μl of the solution in each group was evaluated for its fluorescence intensity (GOLMAX-MULTI PLUS, Promega, USA).

For data presentation, the coaggregation results of *S. mutans* derivatives/GBS-SI101 groups and GBS-SI101 only group are shown as the percentage of the fluorescence signal compared with the fluorescence intensity of the wild-type *S. mutans*/GBS-SI101 control group. Three replicates were performed for this assay.

### Growth Competition Assay

Growth competition assay was performed as described previously with modifications (Tong et al., 2007). Overnight cultures of *Streptococcus* species, including *S. mutans, S. sanguinis*, and *S. gordonii*, and GBS-SI101 were adjusted to the same optical density at 600 nm (OD_600_ ~ 1). Ten-microliter aliquots of GBS-SI101 and 10-μl aliquots of each *Streptococcus* species were, sequentially or simultaneously, inoculated adjacently on SHI agar plates such that the bacterial spots almost touched each other. In the sequential inoculation groups, species that were inoculated first were grown anaerobically overnight, followed by the inoculation of the other species. The plates were incubated anaerobically for 24 h. Growth inhibition was assessed by the presence of a proximal zone of inhibition.

### Viability Assay in Spent Culture Media (Spent Medium Assay)

GBS-SI101 and *Streptococcus* species, including *S. mutans, S. sanguinis*, and *S. gordonii*, were cultured independently in THB. After overnight cultivation under anaerobic conditions, the supernatants were collected as spent media and filter-sterilized.

First, we examined whether diffusible metabolites of oral streptococci affected the viability of GBS-SI101. GBS-SI101 was grown in different media, including PBS (control), GBS-SI101 spent medium (control), *S. mutans* spent medium, *S. sanguinis* spent medium, and *S. gordonii* spent medium. Each group was supplemented with an equal volume of fresh THB. Viability counts (CFU/ml) were monitored for GBS-SI101 in different media after 24 h incubation under anaerobic conditions. Three replicates were performed for this assay.

Second, we examined whether diffusible metabolites of GBS-SI101 affected the growth of *Streptococcus* species. Each of the *Streptococcus* species was inoculated into its own spent medium and GBS-SI101 spent medium. Each group was supplemented with an equal volume of fresh THB. Viability counts (CFU/ml) were monitored for each of the *Streptococcus* species in each spent medium after 24 h incubation under anaerobic conditions. Three replicates were performed for this assay.

### Statistical Analysis

Statistical significance (*p* <0.05) of differences was analyzed by One-Way analysis of variance (ANOVA) with *post-hoc* Dunnett's test.

## Results

### GBS From Saliva Microbiota Adhered to *S. mutans* Biofilm

**Figure 1:**
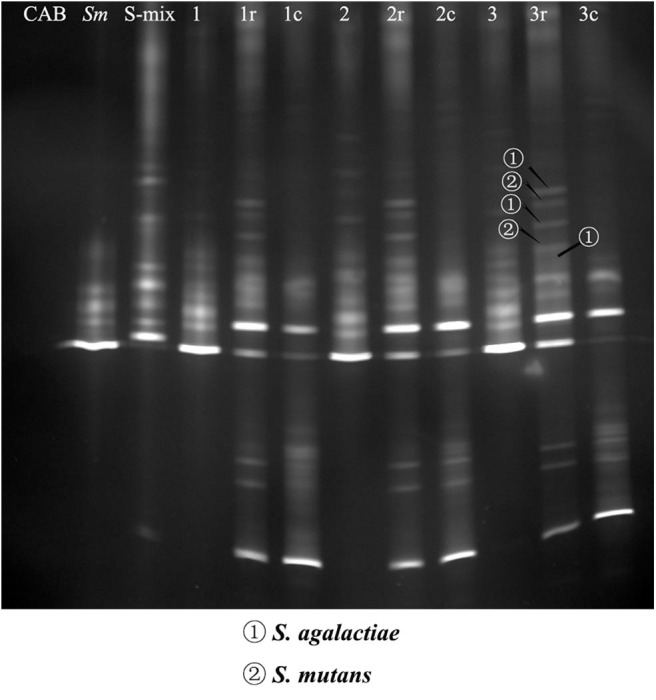
PCR-DGGE profiles of co-adhering mixtures. S-mix was overlaid onto *S. mutans* biofilms and allowed to stand for 1 h (Group 1), 2 h (Group 2), and 3 h (Group 3). Unbound cells were washed. Then, the co-adhering mixtures were collected either immediately or after overnight regrowth in fresh SHI medium under anaerobic conditions (Groups 1r, 2r, and 3r). In the control groups, CAB, instead of S-mix, was overlaid onto *S. mutans* biofilm, and the biofilm was collected after incubation times of 1 h (Group 1c), 2 h (Group 2c), and 3 h (Group 3c). The first three lanes on the left represent the blank control (CAB), *S. mutans* (*Sm*) and species profile of S-mix (S-mix), respectively. DNA bands of interest were excised, and DNA samples were recovered and sequenced. *S. agalactiae* and *S. mutans* were identified by sequencing and are indicated with arrows.

The pull-down assay has been used successfully to identify bacterial species that exhibit physical interactions with oral bacteria (Guo et al., 2015). In this study, we used this assay, together with the PCR-DGGE technique, to detect saliva isolates that could physically bind to *S. mutans*. Bands were relatively blurry in co-adhering mixtures, possibly due to the short incubation time and the low abundance of bacteria. Bands were of higher intensity in the co-adhering mixtures that were regrown overnight. DNA samples were recovered from the DGGE bands of interest, and GBS-SI101 was identified by sequencing. Our results showed that GBS-SI101 could be pulled down by *S. mutans* biofilms *in vitro* (Figure 1).

**Figure 2:**
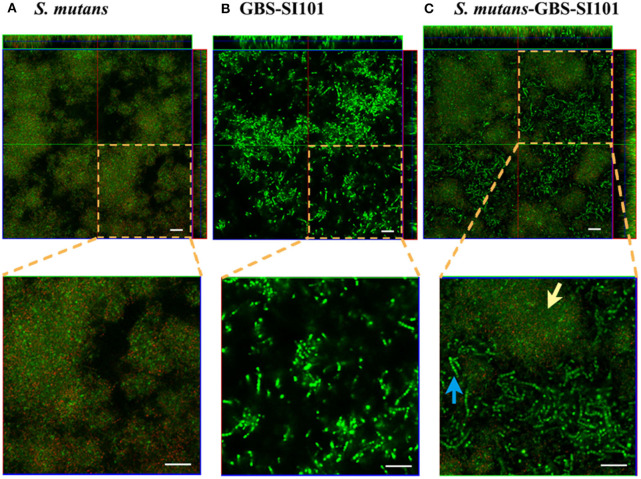
Monospecies and dual-species biofilms visualized by CLSM. GBS-SI101 was overlaid onto 6-well culture plates with pre-coated pooled saliva proteins or pre-existing *S. mutans* biofilms. The plates were incubated anaerobically overnight. Biofilms were labeled with SYTO 9 displaying green in CLSM imaging. Biofilm *S. mutans* cells were further specifically labeled with an anti-*S. mutans* monoclonal antibody SWLA1–IgG2a, which was later attached with Alexa Fluor 633-conjugated goat anti-mouse IgG, displaying red in CLSM imaging. **(A)** *S. mutans* biofilm; **(B)** GBS-SI101 biofilm; **(C)** *S. mutans* and GBS-SI101 dual-species biofilm. The green cluster (yellow arrow) represents *S. mutans* microcolonies where monoclonal antibody SWLA1–IgG2a failed to penetrate. The blue arrow points to GBS-SI101 within the dual-species biofilm. The scale bar represents 20 μm.

CLSM imaging (Figure 2A) showed that *S. mutans* formed monospecies biofilms. The *S. mutans* cells, labeled with specific antibodies, are displayed in red. The far-red dye failed to penetrate some dense microcolonies in *S. mutans* biofilms. The enlarged images in Figures 2A,B showed that the cell sizes of GBS-SI101 were visually larger than those of *S. mutans***.** CLSM imaging of the dual-species biofilm (Figure 2C) confirmed GBS integration into the *S. mutans* biofilm.

### GtfB and GtfC of *S. mutans* Were Involved in Coaggregation With GBS-SI101

**Figure 3:**
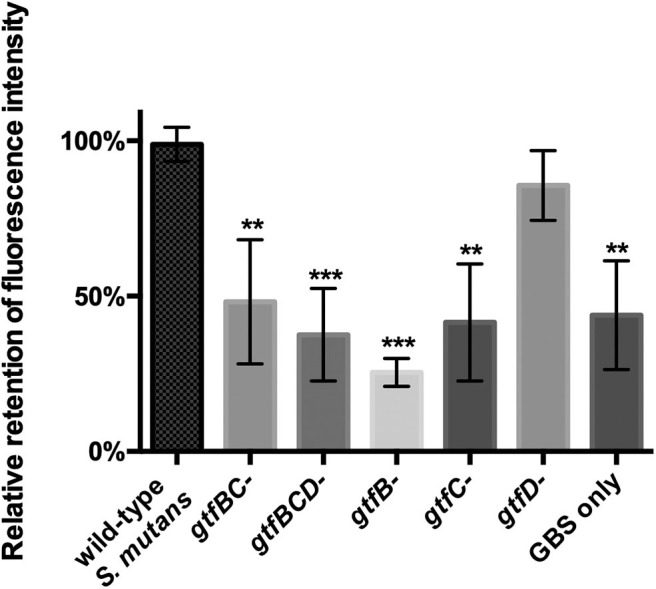
Coaggregation between GBS-SI101 and *S. mutans gtf*-deficient derivatives. *gtfB*-, *gtfC*-, *gtfD*-, *gtfBC*-, and *gtfBCD*-deficient strains, as well as the parent strain of *S. mutans*, were used to coaggregate with SYTO 9-labeled GBS-SI101. The coaggregation result was shown as the ratio of fluorescence intensity retained in co-aggregates to that in the wild-type *S. mutans*/GBS-SI101 pairs. Data represent the mean ± S.D. of the results of three independent experiments. The asterisk indicates a significant difference between each testing group and the parent strain *S. mutans*/GBS-SI101 group (*p* < 0.05).

Glucosyltransferases (Gtfs) are mainly responsible for synthesizing EPS in *S. mutans. S. mutans* utilizes EPS to facilitate intercellular and interspecies adhesion (Koo et al., 2010). Three *gtf* genes expressing Gtfs activity in *S. mutans*, including *gtfB, gtfC*, and *gtfD*, have been identified (Kuramitsu, 1993). The results from the fluorescence-based coaggregation assay showed that, compared with *S. mutans* parent strain, *gtfB*-, *gtfC*-, *gtfBC*-, and *gtfBCD*-deficient strains displayed significantly reduced levels of interspecies binding with GBS-SI101 (Figure 3; *p* < 0.05). The *gtfD*-deficient strain of *S. mutans*, similar to the parent strain, demonstrated high levels of interspecies coaggregation with GBS-SI101 (Figure 3). The results indicated that lack of either *gtfB-* or *gtfC-*encoded functions impaired *S. mutans*' ability to physically bind to GBS-SI101.

### The Growth of GBS-SI101 Was Not Affected by Oral Streptococci

**Figure 4:**
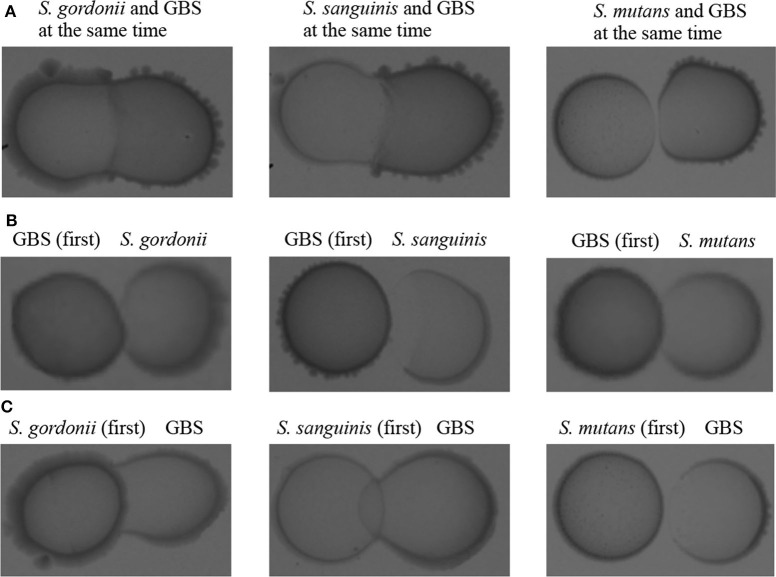
Growth competition between GBS-SI101 and three different *Streptococcus* species. **(A)** GBS-SI101 and oral streptococci were inoculated simultaneously under anaerobic conditions. **(B)** GBS-SI101 was inoculated first and allowed to grow anaerobically overnight. **(C)** Oral streptococci were inoculated first and allowed to grow anaerobically overnight.

**Figure 5:**
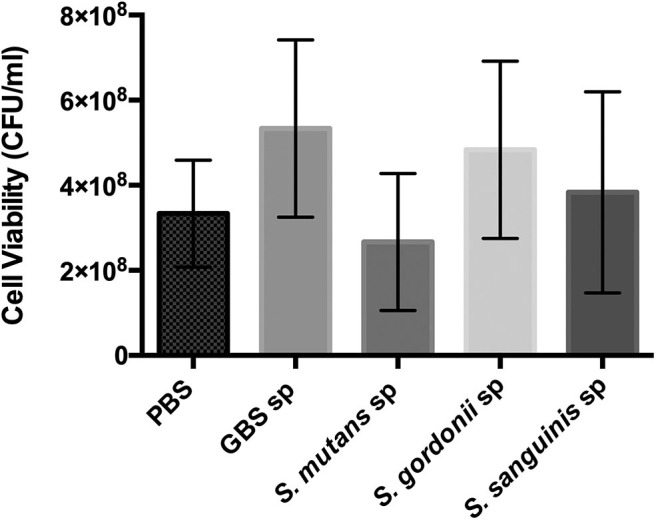
Viability of GBS-SI101 in *Streptococci-*spent media. GBS-SI101 was inoculated into PBS (control), and the spent medium (sp) of GBS-SI101 (control), *S. mutans, S. gordonii*, and *S. sanguinis*, respectively. Each group was supplemented with an equal volume of fresh THB. Viability counts (CFU/ml) were monitored for GBS-SI101 in all six groups after 24 h incubation under anaerobic conditions. Data represent the mean ± S.D. of the results of three independent experiments.

**Figure 6:**
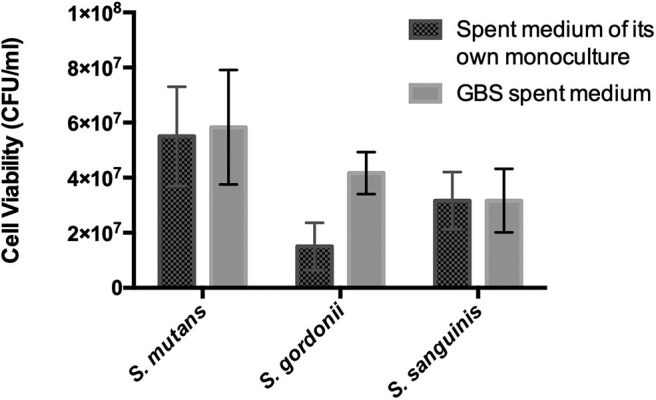
Viability of *Streptococcus* species in GBS-spent media. Each *Streptococcus* species was cultured in the spent medium of its own and GBS spent medium. An equal volume of fresh THB was added to each spent medium. Viability counts (CFU/ml) of the *Streptococcus* species in their own spent medium or the GBS spent medium were monitored after 24 h incubation under anaerobic conditions. Data represent the mean ± S.D. of the results of three independent experiments.

Oral streptococci are predominant microorganisms in the oral microbiota (Jakubovics et al., 2014). Our study further assessed the potential growth competition between GBS-SI101 and common oral streptococci. GBS-SI101 was inoculated close to different streptococci species on SHI agar plates and grown anaerobically for 24 h. A clear growth-inhibition zone of *S. sanguinis* was observed when GBS-SI101 was inoculated first, however, no inhibition on GBS-SI101 was monitored when *S. sanguinis* was inoculated first. Meanwhile, no inhibition zone was observed for either strain when they were inoculated simultaneously. The results suggested that the sequence of inoculation influenced the growth antagonism of GBS-SI101 to *S. sanguinis*. Growth-inhibition zones were not observed in other *Streptococcus* spp./GBS-SI101 pairs, indicating that *S. mutans* and *S. gordonii* did not inhibit the growth of GBS-SI101 on SHI agar plates, and vice versa (Figure 4). We further investigated the effects of diffusible metabolites on bacterial growth by adopting the spent medium assay. The results showed that metabolites produced by tested *Streptococcus* species had nonsignificant effects on the viability of GBS-SI101 (Figure 5). Similarly, GBS-SI101 spent media did not significantly affect the viability of *S. mutans, S. gordonii*, and *S. sanguinis* compared to their own spent media control (Figure 6).

## Discussion

In the complex oral microbial community, interspecies coaggregation creates close spatial proximity, promoting close intercellular communication and impacting polymicrobial pathogenesis (Hansen et al., 2007). In addition, intergeneric interactions may influence the establishment of highly structured multispecies oral biofilms (Rickard et al., 2003). In this study, our results showed that GBS-SI101, derived from saliva samples, adhered to *S. mutans* directly. Enzymes associated with EPS biosynthesis, including GtfB and GtfC, could play a significant role in the physical interplay between *S. mutans* and GBS-SI101.

*S. mutans* forms stable bonds with other oral bacteria or salivary pellicles via sucrose-dependent or sucrose-independent mechanism (Koga et al., 1986; Cvitkovitch et al., 2003). Gtfs are responsible for converting sucrose to glucans and play a critical role in sucrose-dependent adhesion (Krzysciak et al., 2014). Our results showed that GtfB and GtfC of *S. mutans*, which were mainly responsible for the synthesis of water-insoluble glucans (Aoki et al., 1986; Hanada and Kuramitsu, 1988), were involved in dual-species adhesion between *S. mutans* and GBS-SI101. Loss of either *gtfB-* or *gtfC-*encoded functions impaired the binding of *S. mutans* with GBS-SI101, which is consistent with a previous study associating GtfB and GtfC with microbial adherence and biofilm formation in *S. mutans* (Koo et al., 2010).

EPS production by Gtfs enhances the formation of a spatially distinct 3-dimensional matrix, increasing the creation of highly cohesive *S. mutans* biofilms (Xiao et al., 2012). In parallel, *S. mutans* cells within biofilms can convert fermentable carbohydrates (such as sucrose) into acids, resulting in a low pH microenvironment (Xiao et al., 2012). The continuous low pH below a critical point (5.5) will promote enamel demineralization and consequent development of cavitation (Takahashi and Nyvad, 2008). Low pH environments also limit the growth of many acid-sensitive oral commensals, promoting the development of *S. mutans*-dominated biofilms (Abranches et al., 2018).

When GBS adheres to *S. mutans*, GBS will likely be exposed to acidic microenvironments. Our results showed that neither the close dual-species interactions on SHI agar plates nor the diffusible metabolites of planktonic *S. mutans* interfered with the growth of GBS-SI101. GBS is a commensal bacterium of gastrointestinal and vaginal tracts and favors colonization in the acidic vaginal mucosa (pH 4.0 ± 0.5) (Verani et al., 2010; Shabayek and Spellerberg, 2017). Since GBS can survive and cause infections in the acidic genital tracts, it would not be surprising that GBS could thrive in the *S. mutans*-dominant acidic environment. Early studies verified enhanced adherence of GBS to alveolar epithelial cells (A549 cell line) at low pH conditions (Zawaneh et al., 1979; Tamura et al., 1994). D'Urzo et al. (2014) studied 389 GBS isolates and found that acidified culture media can increase GBS biofilm formation. To survive the acidic microenvironments, GBS has possessed several mechanisms similar to *S. mutans*, such as arginine deiminase system (ADI), proton pumps, and acid tolerance response (ATR) (Shabayek and Spellerberg, 2017; Lemos et al., 2019). Hence, the acid-generating *S. mutans* might offer a favorable niche for GBS to survive in the oral cavity.

In addition to generating acids, *S. mutans* can produce bacteriocins (mutacins) to impair the growth of nearby bacterial species (Qi et al., 2001). Bacteriocins are antimicrobial peptides (AMPs) produced by bacteria and are classified as non-lantibiotics or lantibiotics (Riley and Wertz, 2002). Lantibiotics are post-translationally modified cationic AMPs and contain unusual amino acids (dehydroalanine and dehydrobutyrine) (Draper et al., 2015).

Under planktonic conditions, *S. mutans* UA140 produces nonlantibiotic mutacin IV, which mainly targets group A streptococci and mitis group streptococci (Qi et al., 2001). The results of the spent medium assay showed that metabolites in the *S. mutans* spent medium nonsignificantly affected the growth of GBS-SI101. Planktonic GBS cells are faced with various challenges, but they have developed certain coping strategies. One possible approach for GBS to address toxic diffusible metabolites such as mutacins might be the production of capsular polysaccharides (CPSs). The CPSs of GBS attach to the cell wall peptidoglycan and form a capsule layer covering the cell surface (Deng et al., 2000). Studies have suggested that surface-bound CPSs may endow bacterial species with resistance to AMPs (Campos et al., 2004; Spinosa et al., 2007; Llobet et al., 2008). Campos et al. (2004) showed that CPSs protect *Klebsiella pneumoniae* by limiting interactions between AMPs and the cell surface. Llobet et al. (2008) also demonstrated that anionic CPSs attract cationic AMPs to reduce the number of peptides binding to the bacterial surface.

When grown on a culture plate, *S. mutans* UA140 produces the cationic lantibiotic mutacin I, which is active against various gram-positive bacteria (Qi et al., 2001; Nicolas et al., 2007). Khosa et al. (2016) discovered that GBS expresses a resistance protein called *Sa*NSR. By cleaving off specific amino acids, *Sa*NSR can reduce the antimicrobial activity of a lantibiotic produced by *Lactococcus lactis* (Khosa et al., 2016). In addition, the GBS cell wall comprises a thick peptidoglycan layer as well as glycerol-phosphate polymers called lipoteichoic acid (LTA). Saar-Dover et al. (2012) showed that D-alanylation of LTAs in GBS results in reduced flexibility and permeability of the cell wall, leading to enhanced resistance to cationic AMPs. GBS has the potential to counteract the antimicrobial effects of lantibiotics, but further studies are needed to elucidate the specific responses of GBS to lantibiotics produced by *S. mutans*.

A healthy and balanced oral microbial community is often associated with greater proportions of commensal streptococci from mitis and sanguinis groups and lower proportions of *S. mutans* (Abranches et al., 2018). *S. gordonii* and *S. sanguinis* are both predominant oral colonizers that modulate the balanced structure in the oral microbiota (Abranches et al., 2018). The generation of oxidative stress gives these species a competitive edge over some oral pathogens. Kreth et al. (2008) reported that both *S. gordonii* and *S. sanguinis* produced significant amounts of hydrogen peroxide (H_2_O_2_) to repress the growth of *S. mutans*, although the production of H_2_O_2_ was reduced under anaerobic conditions. Liu et al. (2011) showed that *S. gordonii* biofilms grown under aerobic conditions generated a steady level of 1.4 mM H_2_O_2_ at 100 μm above the biofilm surface, and the concentration of H_2_O_2_ decreased at a greater distance.

GBS, which binds to *S. mutans*, might also experience selective pressure from dominant commensal streptococci. Our results showed that *S. gordonii* or *S. sanguinis* imposed few inhibitory effects on the growth of GBS. Poyart et al. (2001) demonstrated that superoxide dismutase (SodA) of GBS is crucial in GBS resistance to high levels of H_2_O_2_ (up to 20 mM). Other studies also showed that GBS, utilizing MntH (Mn21/Fe21 transporter) and NADH oxidase, can survive exposure to high levels of oxidative stress (Shabayek et al., 2016; Korir et al., 2018). Therefore, GBS might be able to endure oxidative stress from these oral commensals. Interestingly, in the growth competition assay, we found that GBS-SI101 could inhibit *S. sanguinis* in an inoculation sequence-dependent manner, and further studies are warranted to explore the mechanism of this phenomenon.

## Conclusion

The focus of this study was to discover a new binding partner of *S. mutans* and investigate interactions between them. We identified for the first time the interspecies cell-cell contact between *S. mutans* and GBS-SI101, which typically resides in the gastrointestinal and vaginal tracts. GtfB and GtfC of *S. mutans*, which are responsible for EPS biosynthesis, played essential roles in this interspecies coaggregation. In addition, several oral streptococcus species used in this study barely impaired the growth of GBS-SI101. Instead of being a transient species in the oral cavity, GBS-SI101 might bind to *S. mutans* and overcome interspecies growth competition from commensal streptococci, integrating into the *Streptococcus*-associated polymicrobial community.

Given the limitation of an *in vitro* study, our results cannot fully represent the sophisticated situations in the oral microbial community. The real process *in vivo* during the integration of GBS into *Streptococcus*-associated oral multispecies community will be far more complex and may involve extensive intergeneric and host-GBS interactions. Nevertheless, our study investigated the *S. mutans*-GBS-SI101 interactions and provided evidence for a possible strategy used by GBS to colonize and persist within the oral cavity. Synergistic interactions between the two opportunistic pathogens might affect the balanced oral microbiota and increase the risk of oral infections.

## Data Availability Statement

All datasets generated for this study are included in the article/supplementary material.

## Ethics Statement

The studies involving human participants were reviewed and approved by Ethical Committee of Guanghua School of Stomatology, Hospital of Stomatology, Sun Yat-Sen University, Guangzhou, PR China. The patients/participants provided their written informed consent to participate in this study.

## Author Contributions

WS and LG designed and performed the experiments. LG, JiaL, and TL analyzed the data. LG and TL wrote the manuscript. All authors revised the manuscript critically and approved the final manuscript.

## Conflict of Interest

The authors declare that the research was conducted in the absence of any commercial or financial relationships that could be construed as a potential conflict of interest.

## Abbreviations

AMPs: Antimicrobial peptides
*C. albicans*: *Candida albicans*
CPSs: capsular polysaccharides
GBS-SI101: saliva isolate *Streptococcus agalactia*
CAB: coaggregation buffer
CLSM: confocal laser scanning microscopy
ECC: early childhood caries
EPS: extracellular polysaccharides
*Fnp*: *Fusobacterium nucleatum ssp*. polymorphum
Gtfs: glucosyltransferases
LTA: lipoteichoic acid
NAM: N -acetylmuramic acid
PBS: phosphate-buffered saline
S-mix: saliva-derived microbiota
*S. agalactiae*: Streptococcus agalactiae
GBS: also known as Group B *Streptococcus*
*S. gordonii*: *Streptococcus gordonii*
*S. mutans*: *Streptococcus mutans*
*S. sanguinis*: *Streptococcus sanguinis*
SodA: superoxide dismutase
THB: Todd-Hewitt Broth.
